# Gut microbiome enterotypes drive divergent lactation performance in dairy goats through host-microbe metabolic cross-talk

**DOI:** 10.1128/spectrum.01367-25

**Published:** 2025-09-26

**Authors:** Dangdang Wang, Qingyan Yin, Guangfu Tang, Li Sun, Junjian Yu, Yangchun Cao, Junhu Yao

**Affiliations:** 1 College of Animal Science and Technology, Northwest A&F University 12469, Yangling, Shaanxi, China; 2 State Key Laboratory for Diagnosis and Treatment of Severe Zoonotic Infectious Diseases, Key Laboratory for Zoonosis Research of the Ministry of Education, Institute of Zoonosis, and College of Veterinary Medicine, Jilin University 12510, Changchun, Jilin, China; 3 National Center of Technology Innovation for Dairy, Inner Mongolia Dairy Technology Research Institute Co. Ltd. 701580 , Hohhot, Inner Mongolia, China; Nanjing Agricultural University, Nanjing, China

**Keywords:** dairy goat, gut microbiome, enterotype, milk production, co-occurrence network, functional prediction

## Abstract

The gut microbiome plays a pivotal role in modulating nutrient metabolism and lactation efficiency in ruminants, yet its functional stratification into enterotypes and implications for dairy goats remain unexplored. Here, we identified two distinct gut microbiome enterotypes in 134 lactating dairy goats: TR-cluster (*n* = 43) was enriched in *Turicibacter*, *Romboutsia*, and *Clostridium sensu stricto 1*, while CO-cluster (*n* = 91) exhibited higher abundance of *Christensenellaceae R-7* and *Oscillospiraceae UCG-005*. Goats in the TR-cluster exhibited higher milk yield, fat-corrected milk yield, and milk fat yield, along with increased concentrations of total volatile fatty acids (VFA), including acetate, propionate, and butyrate in the gut, as well as elevated serum glucose and total bile acids levels. Despite lower microbial diversity, the TR-cluster was functionally enriched in metabolic pathways related to energy metabolism, amino acid metabolism, and lipid biosynthesis, whereas the CO-cluster showed enrichment in membrane transport and genetic information processing. Co-occurrence network analysis revealed distinct microbial interaction patterns and keystone taxa, with the TR-cluster displaying more complex inter-module connectivity. Moreover, correlation analyses demonstrated that TR-cluster-enriched genera, such as *Turicibacter* and *Romboutsia*, were positively associated with enhanced gut fermentation and improved lactation traits. Overall, these findings reveal that gut microbial enterotypes are strongly linked to gut fermentation and lactation traits in dairy goats, offering novel insights for microbiota-targeted strategies to improve ruminant productivity.

**IMPORTANCE:**

Dairy goats are critical for global milk production, yet the gut microbiome-driven mechanisms that underlie individual differences in lactation performance remain largely unknown. To our knowledge, this is the largest cohort study to date that comprehensively characterizes gut microbiome enterotypes in lactating dairy goats and establishes their impact on gut fermentation, host metabolism, and milk production. We identified two distinct enterotypes with different microbial signatures, functional capacities, and ecological structures. Notably, the enterotype dominated by *Turicibacter*, *Romboutsia*, and *Clostridium sensu stricto 1* was associated with improved lactation performance, enhanced VFA production, and beneficial metabolic profiles. The identification of keystone taxa and enterotype-specific microbial interactions offers a new perspective on host-microbiome relationships in ruminants. Our findings lay the foundation for precision microbiome management and targeted interventions to enhance the health and productivity of dairy animals.

## INTRODUCTION

The gut microbiome, a dynamic ecosystem of trillions of microorganisms, plays a pivotal role in regulating host metabolism, nutrient utilization, and immune function (1–3). In ruminants, the gut microbiome serves as a critical interface for nutrient conversion, contributing to volatile fatty acid (VFA) production, nitrogen metabolism, and the bioavailability of precursors essential for milk synthesis (4, 5). Previous studies in dairy cows and goats have revealed that specific microbial taxa are associated with inter-individual variations in feed efficiency and milk composition (6–8). The gut microbiota functions as an interactive community, where microbial relationships shape its structure and impact host performance. Identifying key taxa and their interactions is crucial to understanding their role in dairy goats.

Accumulating evidence indicates that microbial community configurations are not randomly distributed but can be classified into distinct clusters known as enterotypes-host-associated microbiota profiles that are relatively stable and functionally differentiated (9–11). Enterotype-specific microbial networks have been linked to variations in methane emissions, feed efficiency, and growth rates in dairy cows and young goats (12–14). However, the existence and implications of gut enterotypes remain poorly characterized in dairy goats. Their potential associations with lactation performance, milk composition, and host metabolism have yet to be explored. Understanding the existence and characteristics of enterotypes in dairy goats could provide valuable insights into the complex interactions between the gut microbiome and host physiology and potentially lead to the development of targeted strategies to improve dairy goat productivity.

In this study, we conducted a comprehensive enterotype-based analysis of the gut microbiota in 134 lactating dairy goats using 16S rRNA gene sequencing. We aimed to: identify distinct gut enterotypes based on microbial community composition; explore the associations between enterotypes and lactation performance, milk composition, and serum metabolic markers; and investigate microbial interaction networks and predict functional capabilities using PICRUSt2. Our findings provide novel insights into the role of gut microbiome enterotypes in modulating dairy goat productivity, offering a foundation for microbiota-driven strategies to enhance milk production efficiency.

## RESULTS

### Identification and characterization of gut microbiome enterotypes in lactating dairy goats

**Fig 1:**
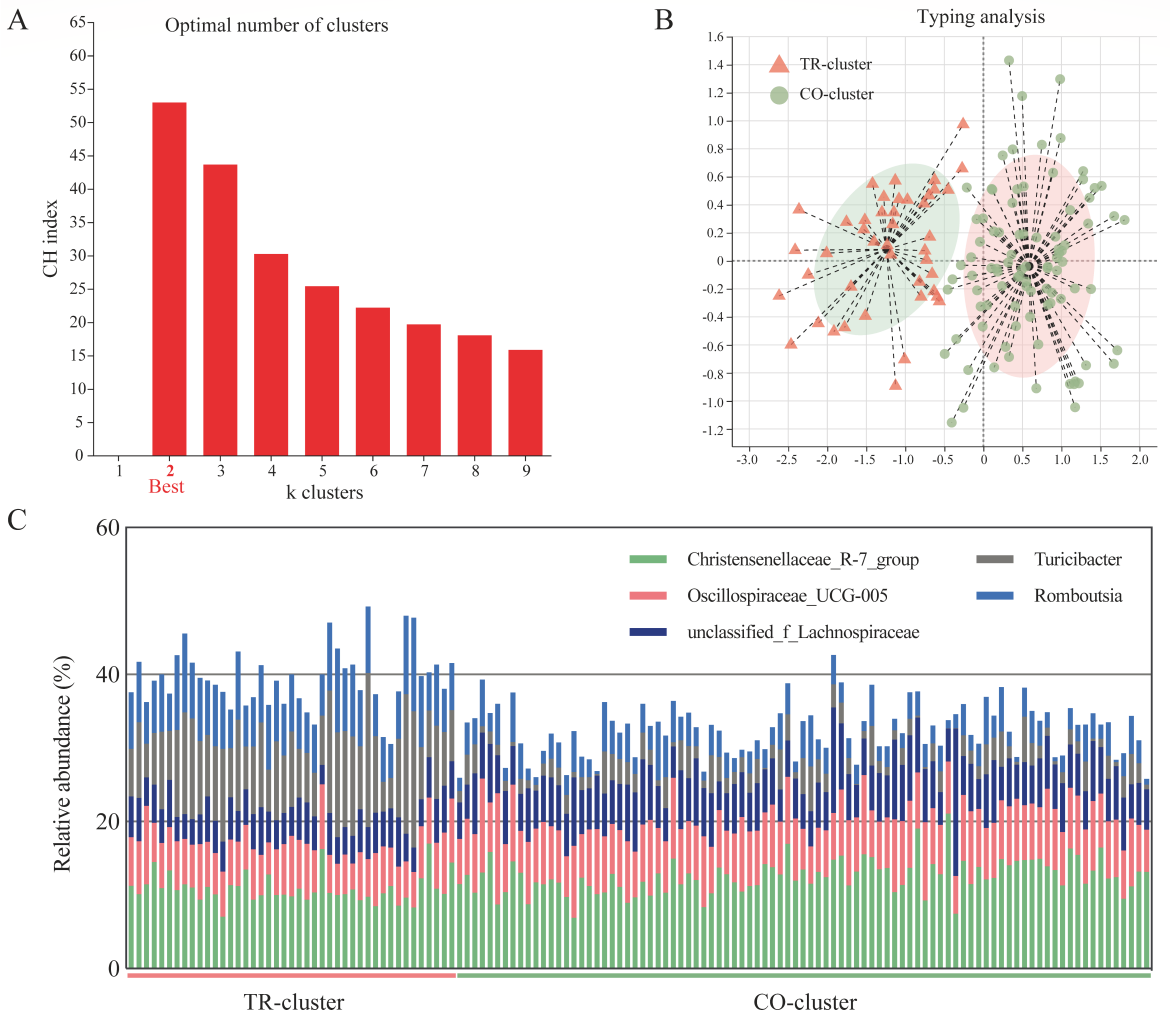
Gut enterotype analysis in dairy goats (*n* = 134). (**A**) CH index of gut enterotype robustness. (**B**) Principal coordinate analysis (PCoA) plot of gut enterotypes based on Jensen-Shannon distance. (**C**) Histogram showing the relative abundance of *Christensenellaceae R-7 group*, *Oscillospriaceae UCG-005*, unclassified *Lachnospiraceae*, *Turicibacter*, and *Romboutsia* in two enterotypes.

The enterotype analysis of the gut microbiome in lactating dairy goats (*n* = 134) is presented in Fig. 1. Using the Calinski-Harabasz (CH) index and partitioning around medoids, we determined the optimal number of clusters to be two (*k* = 2), indicating the most robust microbial partitioning (Fig. 1A). Principal coordinate analysis (PCoA) based on the Jensen-Shannon distance at the genus level revealed clear separation between the two enterotypes (Fig. 1B). As shown in Fig. 1C, TR-cluster is distinguished by *Turicibacter* and *Romboutsia* (*n* = 43), whereas CO-cluster is distinguished by Christensenellaceae R-7 group and Oscillospiraceae UCG-005 (*n* = 91).

### Lactation performance and milk composition in different enterotypes

**Fig 2:**
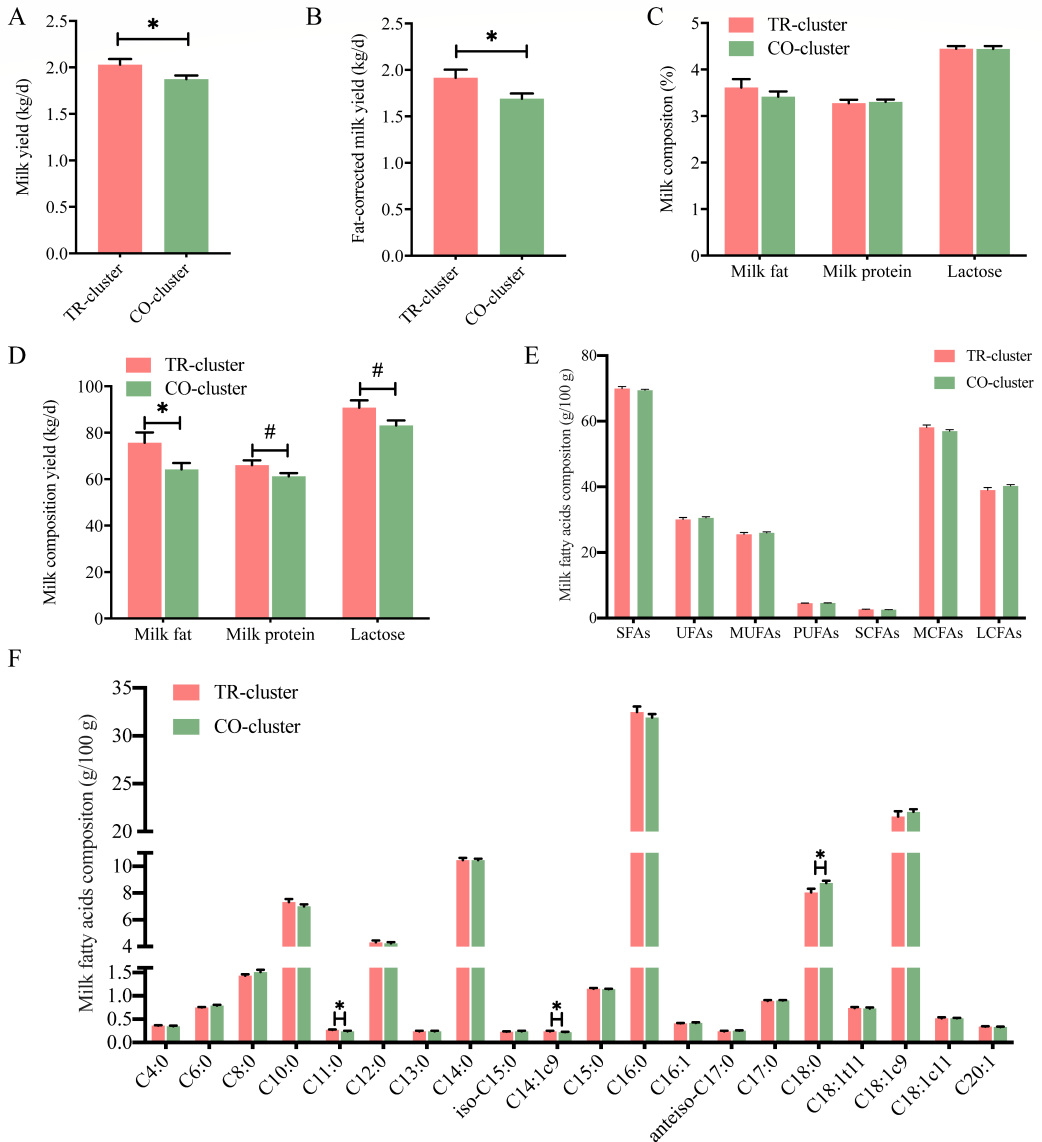
Differences in milk production and composition between TR-cluster and CO-cluster dairy goats. (**A**) Milk yield. (**B**) Fat-corrected milk yield, fat-corrected milk yield =  0.4*M  +  15*F; M, non-standard milk weight (kg); F, fat content of non-standard milk (kg). (**C**) Proportions of milk components, (**D**) yield of milk components, (**E**) major milk fatty acid groups, and (**F**) individual milk fatty acids composition. SFA, saturated fatty acids; UFA, unsaturated fatty acids; MUFA, monounsaturated fatty acids; PUFA, polyunsaturated fatty acids; SCFA, short-chain fatty acids; MCFA, medium-chain fatty acids; LCFA, long-chain fatty acids. Data are presented as mean ± SE. *P < 0.05, ^#^0.05 < *P* < 0.10.

The lactation performance and milk composition of goats in the TR-cluster and CO-cluster were further analyzed. The TR-cluster showed significantly higher milk yield, fat-corrected milk yield, and milk fat yield compared to the CO-cluster (*P* < 0.05, Fig. 2A, B and D). A tendency for increased milk protein and lactose yields was also observed in the TR-cluster (0.05 < *P* < 0.10, Fig. 2D). However, milk fat, protein, and lactose contents did not differ between the two clusters (*P* > 0.05, Fig. 2C).

The milk fatty acid composition is presented in Fig. 2E and F. The TR-cluster displayed significantly higher concentrations of C10:0 and C14:1c9 and a lower concentration of C18:0 compared to the CO-cluster (*P* < 0.05, Fig. 2F). No significant differences were observed between the two clusters in the milk concentrations of saturated fatty acids (SFAs), unsaturated fatty acids (UFAs), monounsaturated fatty acids (MUFAs), polyunsaturated fatty acids (PUFAs), short-chain fatty acids (SCFAs), medium-chain fatty acids (MCFAs), and long-chain fatty acids (LCFAs) (*P* > 0.05, Fig. 2E).

### Gut fermentation and serum biochemical characteristics in different enterotypes

**Fig 3:**
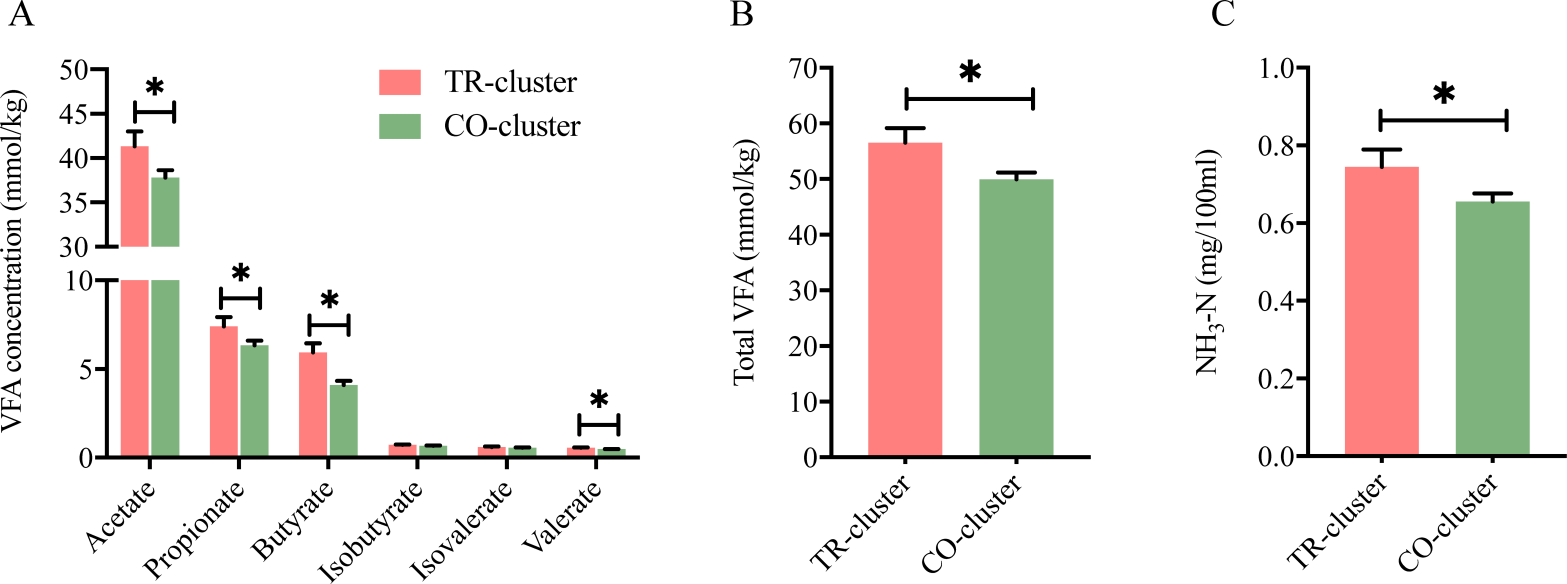
Differences in fecal VFA and NH_3_-N concentrations between TR-cluster and CO-cluster dairy goats. (**A**) Concentrations of individual VFA. (B) VFA concentration. (C) NH_3_-N concentration. Data are presented as mean ± SE. *P < 0.05.

To explore how enterotypes influence host metabolism, we analyzed gut fermentation and serum parameters. Compared to the CO-cluster, the TR-cluster showed higher concentrations of acetate, propionate, butyrate, valerate, and total VFA, along with a greater molar percentage of butyrate but a lower acetate percentage in feces (*P* < 0.05, Fig. 3A and B). Additionally, fecal ammonia nitrogen (NH_3_-N) concentration was significantly higher in the TR-cluster (*P* < 0.05, Fig. 3C).

Regarding serum parameters, the TR-cluster had significantly higher concentrations of globulin, glucose, and total bile acid, but lower levels of albumin and a reduced albumin to globulin ratio (*P* < 0.05, Table 1). There were no significant differences between the enterotypes in serum total protein, urea nitrogen, total cholesterol, high-density lipoprotein cholesterol (HDL-C), or low-density lipoprotein cholesterol (LDL-C, *P* > 0.05, Table 1).

**TABLE 1:** Differences in serum parameters between dairy goats with different enterotypes^*a*^

Item	TR-cluster	CO-cluster	*P*-value
Albumin (g/L)	24.03 ± 0.45	25.39 ± 0.26	0.007
Globulin (g/L)	56.91 ± 1.17	54.44 ± 0.64	0.047
Total protein (g/L)	80.93 ± 1.27	79.82 ± 0.65	0.394
Albumin:Globulin	0.43 ± 0.01	0.47 ± 0.01	0.002
Urea nitrogen (mmol/L)	6.51 ± 0.18	6.48 ± 0.12	0.919
Glucose (mmol/L)	2.89 ± 0.08	2.65 ± 0.05	0.014
Total bile acid (μmol/L)	13.88 ± 0.55	10.22 ± 0.27	0.039
Total cholesterol (mmol/L)	1.90 ± 0.06	1.96 ± 0.05	0.401
Triglyceride (mmol/L)	0.31 ± 0.01	0.31 ± 0.01	0.779
HDL-C (mmol/L)	1.02 ± 0.03	1.07 ± 0.02	0.316
LDL-C (mmol/L)	0.59 ± 0.02	0.62 ± 0.01	0.422

^*a*^ HDL-C, high-density lipoprotein cholesterol; LDL-C, low-density lipoprotein cholesterol. Data are shown as mean ± standard error. TR-cluster (*n *= 43), CO-cluster (*n *= 91).

### Gut microbial diversity, structure, and taxonomic composition in different enterotypes

**Fig 4:**
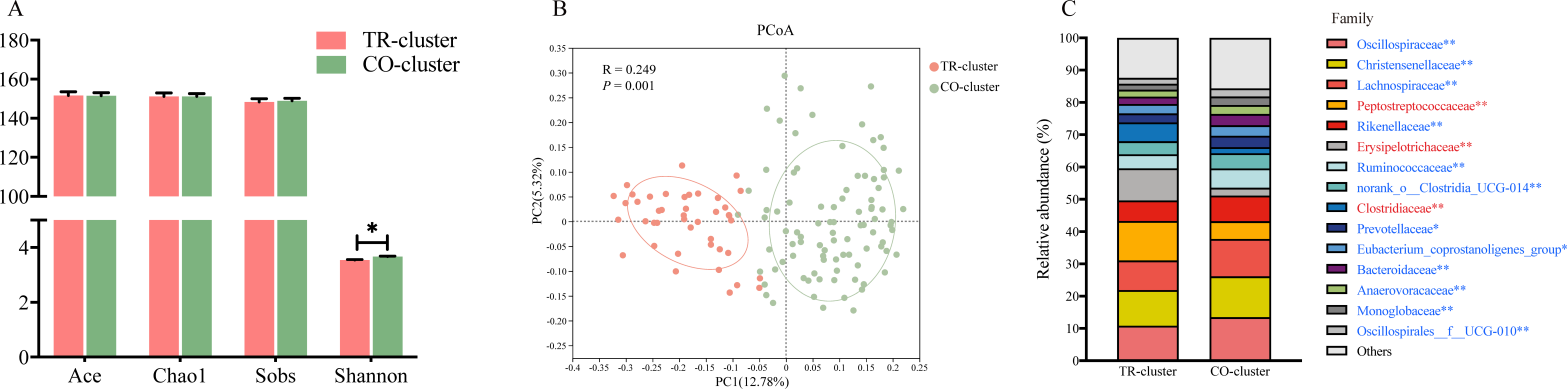
Comparison of gut microbiota richness and diversity between TR-cluster and CO-cluster dairy goats. (**A**) Alpha diversity. (**B**) PCoA plot of gut microbiota based on Bray-Curtis distance. (**C**) Differential microbiota analysis of gut microbiota in two enterotypes at the family level. Bars represent SE. Data are presented as mean ± SE. *P < 0.05, **P < 0.01.

The gut microbiome diversity and bacterial composition of the two enterotypes were further analyzed. The TR-cluster exhibited lower community diversity, as indicated by a significantly lower Shannon index (*P* < 0.05, Fig. 4A). However, no significant differences were observed in richness estimates based on Ace and Chao 1 indices (*P* > 0.05, Fig. 4A). PCoA based on the Bray-Curtis distance demonstrated significant difference in gut microbial structure between the two enterotypes (*P* = 0.001, Fig. 4B).

**Fig 5:**
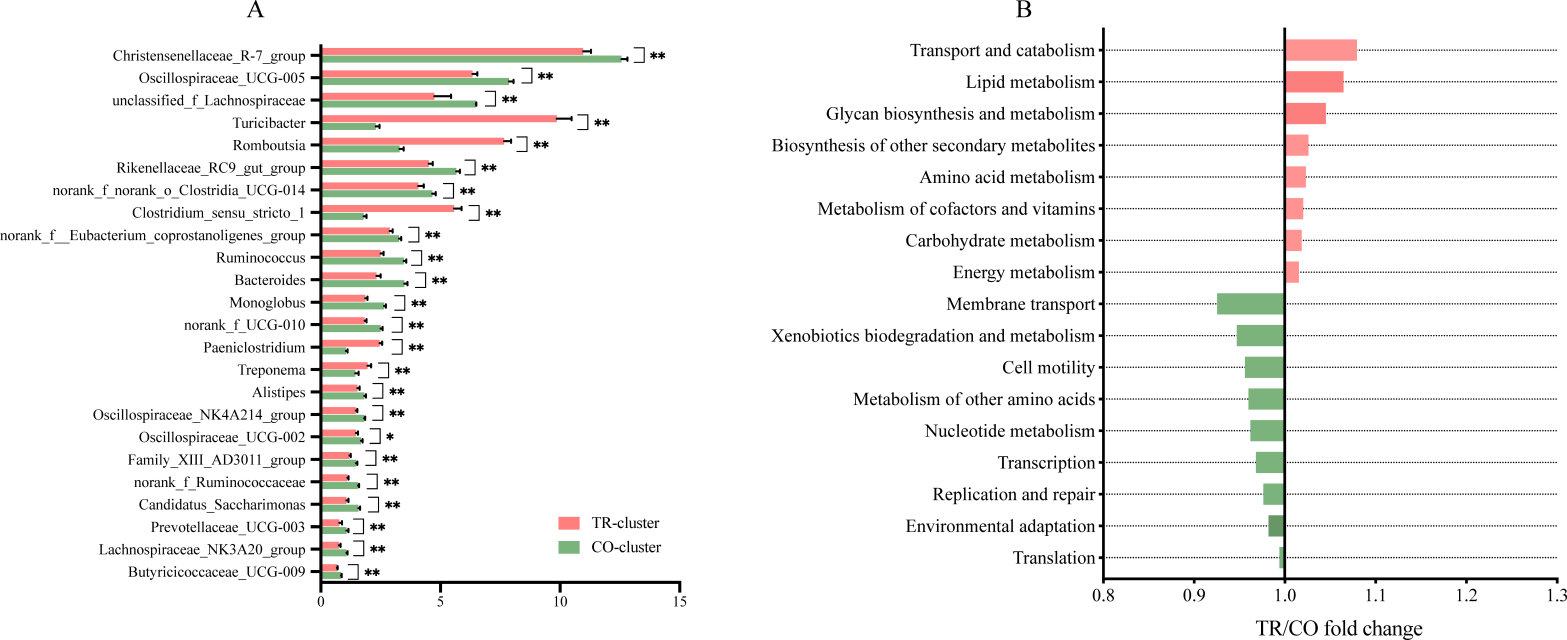
Differences in microbiota composition and function between TR-cluster and CO-cluster dairy goats. (**A**) Relative abundance of gut microbiota at the genera level (%). (**B**) Microbial function prediction differences. Data are presented as mean ± SE. *P < 0.05, **P < 0.01.

The taxonomic differences between the two enterotypes were assessed at the phylum, family, and genus levels (Table 2; Fig. 4C and 5A). Among the top 10 phyla, Firmicutes and Spirochaetota were significantly enriched in the TR-cluster, whereas Bacteroidota, Actinobacteriota, Patescibacteria, Verrucomicrobiota, and Desulfobacterota were more abundant in the CO-cluster (*P* < 0.05, Table 2). At the family level, the abundance of Peptostreptococcaceae, Erysipelotrichaceae, and Clostridiaceae was enriched in the TR-cluster, whereas the abundance of Rikenellaceae and other Firmicutes members, such as Oscillospiraceae, Christensenellaceae, and Lachnospiraceae, was more abundant in the CO-cluster (*P* < 0.05, Fig. 4C).

**TABLE 2:** Comparison of gut microbiota at the phylum level between two enterotypes (%, top 10)^*a*^

Taxon	TR-cluster	CO-cluster	*P*-value
Firmicutes	80.94 ± 0.72	75.44 ± 0.48	<0.001
Bacteroidota	13.90 ± 0.61	18.17 ± 0.46	<0.001
Spirochaetota	1.96 ± 0.16	1.45 ± 0.14	<0.001
Actinobacteriota	1.20 ± 0.08	1.93 ± 0.12	<0.001
Patescibacteria	1.07 ± 0.08	1.57 ± 0.07	<0.001
Verrucomicrobiota	0.29 ± 0.04	0.54 ± 0.04	<0.001
Proteobacteria	0.22 ± 0.03	0.27 ± 0.03	0.437
Cyanobacteria	0.12 ± 0.02	0.14 ± 0.01	0.521
Desulfobacterota	0.09 ± 0.01	0.16 ± 0.01	<0.001
Fibrobacterota	0.10 ± 0.02	0.14 ± 0.02	0.440

^*a*^ Data are shown as mean ± standard error. TR-cluster (*n* = 43), CO-cluster (*n* = 91).

At the genus level, *Turicibacter*, *Romboutsia*, *Clostridium sensu stricto 1*, *Paeniclostridium*, and *Treponema* were enriched in the TR-cluster. Conversely, *Christensenellaceae R-7 group*, *Oscillospiraceae UCG-005*, unclassified *Lachnospiraceae*, and *Rikenellaceae RC9 gut group* were enriched in the CO-cluster (*P* < 0.05, Fig. 5A).

### Microbial function prediction

PICRUSt2 was employed to predict the microbial function profiles based on 16S rRNA gene sequences, using the Kyoto Encyclopedia of Genes and Genomes (KEGG) database for functional annotation (Fig. 5B). In total, 17 significantly different KEGG pathways (level 3) were identified. Pathways related to transport and catabolism, lipid metabolism, glycan biosynthesis and metabolism, amino acid metabolism, carbohydrate metabolism, and energy metabolism were enriched in the TR-cluster (*P* < 0.05, Fig. 5B). Meanwhile, pathways involved in membrane transport, cell motility, metabolism of other amino acids, nucleotide metabolism, transcription, replication, and repair were enriched in the CO-cluster (*P* < 0.05, Fig. 5B).

### Bacterial-bacterial interactions of co-occurrence networks

**Fig 6:**
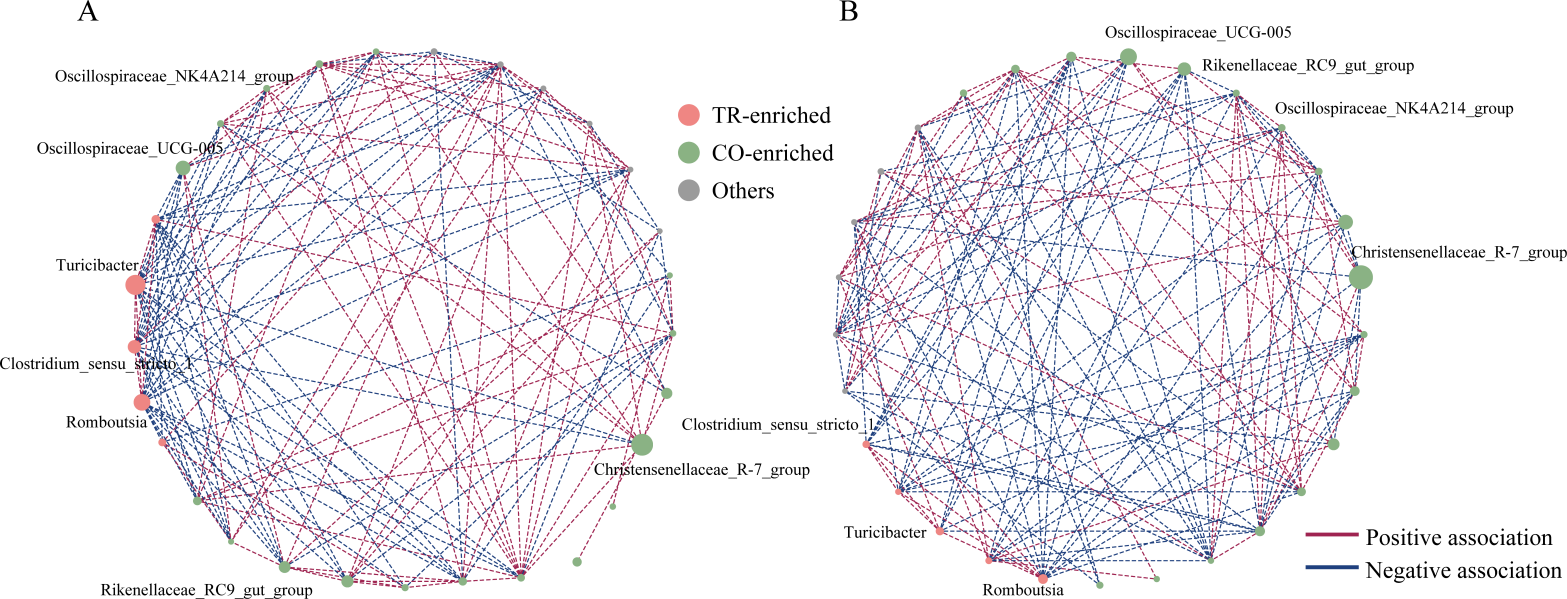
Co-occurrence network of gut microbial genera in TR-cluster and CO-cluster dairy goats. (**A**) Co-occurrence network of genera in TR-cluster and (**B**) in CO-cluster. Only genera with a relative abundance >0.5% and detected in >50% of all samples are included. The color of connection lines indicates correlation type: red for positive and blue for negative (Spearman’s correlation, *P* < 0.05). Node size represents the relative abundance of each genus.

The gut microbial composition and function are largely shaped by intricate bacterial interactions. To assess potential differences between the two enterotypes, we analyzed microbial co-occurrence networks using the top 29 genera (relative abundance >0.5%, present in at least 50% of all samples) (Fig. 6). Distinct interaction patterns were observed between the two enterotypes. Negative correlations were predominantly observed between genera enriched in different clusters, whereas positive correlations were more frequent within the same cluster (Spearman’s correlation, *P* < 0.05, Fig. 6A and B).

In the TR-cluster, *Turicibacter*, *Romboutsia*, and *Clostridium sensu stricto 1* exhibited a greater number of significant correlations, forming 41 significant associations with other bacterial genera. Specifically, these TR-cluster-enriched genera showed significant negative correlations with *Oscillospiraceae NK4A214 group*, *Oscillospiraceae UCG-002*, *Prevotellaceae UCG-004*, *Prevotellaceae UCG-003*, and *Ruminococcus*, which were enriched in the CO-cluster (Spearman’s correlation, *P* < 0.05, Fig. 6A). In contrast, within the CO-cluster, these TR-cluster-enriched genera exhibited fewer associations, forming only 25 connections with other genera (Fig. 6B).

**Fig 7:**
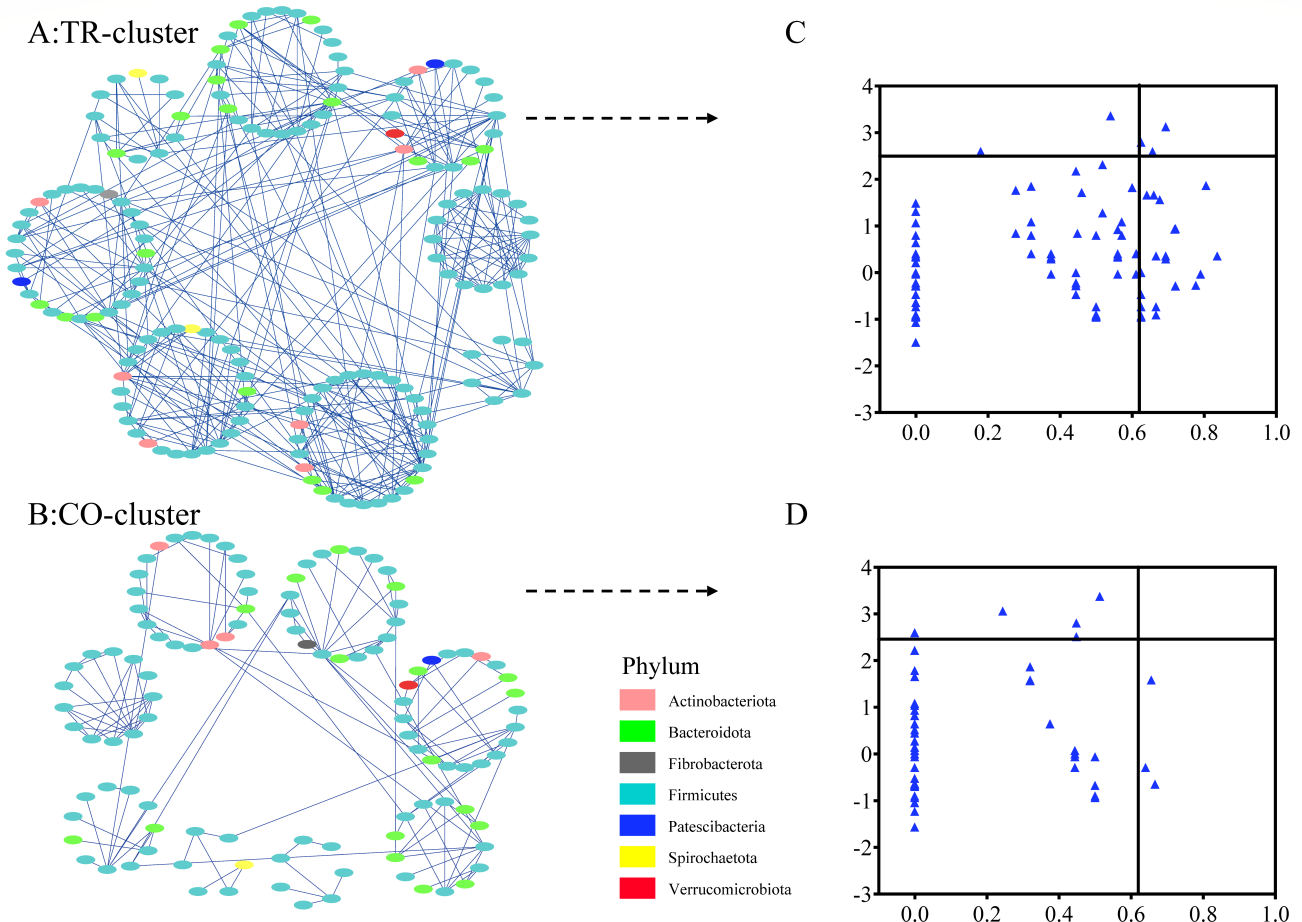
Module analysis of co-occurrence network based on ASV (detected in >50% of all samples) in TR-cluster and CO-cluster dairy goats. (**A**) Co-occurrence network of ASVs in TR-cluster. (**B**) Co-occurrence network of ASVs in CO-cluster. Nodes represent ASVs, with only significant (Pearson’s correlation, *P* < 0.05) relationships indicated by solid lines. (**C**) Scatter plot showing the distribution of ASVs based on network roles in TR-cluster, and (**D**) scatter plot showing the distribution of ASVs based on network roles in CO-cluster. Zi, within-module connectivity; Pi, among-module connectivity.

We further assessed the potential microbial modules and identified the keystone taxa by conducting an RMT-based network analysis in the TR-cluster and CO-cluster (Fig. 7). The analysis revealed that the TR-cluster exhibited more intricate inter-module relationships, whereas the CO-cluster displayed fewer connections among its microbial modules (Fig. 7A and B). In the TR-cluster, three ASVs affiliated with *Ruminococcus*, *Rummeliibacillus*, and *norank Muribaculaceae* demonstrated high *Zi* (>2.5) and *Pi* (>0.62), classifying them as network hubs that highly connected species within its own module and linked several modules together. Two ASVs, belonging to *Christensenellaceae R-7 group* and *Bacillus*, were classified as module hubs, characterized by high *Zi* (>2.5) and low *Pi* (<0.62). Additionally, 24 ASVs, including those affiliated with *Rikenellaceae RC9 gut group*, *Bifidobacterium*, and *Christensenellaceae R-7 group*, exhibited low *Zi* (≤2.5) and high *Pi* (>0.62), functioning as connectors (Fig. 7C). In contrast, the CO-cluster’s network analysis identified five ASVs, including ASV2737 and ASV4519 affiliated with *Turicibacter* and *Christensenellaceae R-7 group*, as module hubs, indicating highly connected species within their own module. Additionally, three ASVs, including ASV2838 annotated to *Christensenellaceae R-7 group*, served as connectors linking multiple modules in the CO-cluster network (Fig. 7D).

### Correlation analysis of gut bacteria with gut fermentation, serum parameters, and milk performance

**Fig 8:**
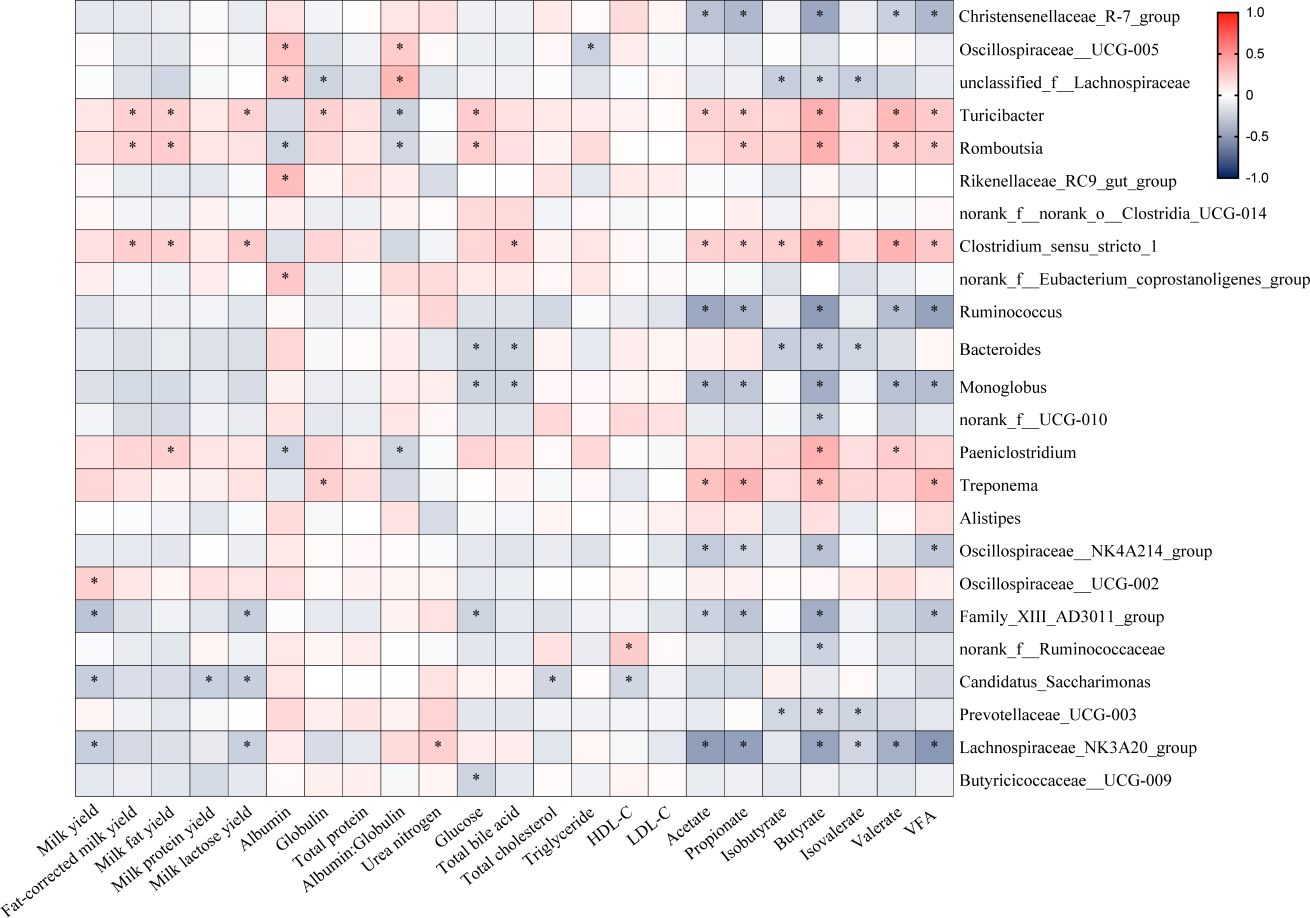
Association analysis between gut genera, milk production traits, serum parameters, and fecal VFA in TR-cluster and CO-cluster dairy goats. Spearman’s correlation, *P < 0.05.

To investigate the relationships of gut microbiota composition with gut fermentation parameters, serum biochemical indices, and milk performance, 24 differential genera (relative abundance >0.5%) identified between the two enterotypes were selected for correlation analysis. As shown in Fig. 8, distinct microbe-trait interaction patterns are observed between genera enriched in the TR-cluster and CO-cluster.

Among the five genera enriched in the TR-cluster, four genera, including *Turicibacter*, *Romboutsia*, *Clostridium sensu stricto 1*, and *Paeniclostridium*, exhibited significant positive correlations with both fat-corrected milk yield and gut butyrate concentration (Spearman’s correlation, *P* < 0.05, Fig. 8). Additionally, the relative abundances of *Turicibacter*, *Clostridium sensu stricto 1*, and *Treponema* were positively associated with the concentrations of gut total VFA, acetate, and propionate (Spearman’s correlation, *P* < 0.05, Fig. 8). In contrast, genera enriched in the CO-cluster, including *Christensenellaceae R-7 group*, *Ruminococcus*, *Monoglobus*, *Oscillospiraceae NK4A214 group*, and *Lacnospiraceae NK3A20 group*, displayed negative correlations with the concentrations of gut total VFA, acetate, propionate, and butyrate (Spearman’s correlation, *P* < 0.05, Fig. 8).

## DISCUSSION

The intricate interplay between the gut microbiome and production performance in dairy goats reflects a complex ecosystem in which microbial composition, functions, and interactions significantly influence host health and production (4, 15, 16). In the present study, we identified and characterized two distinct gut microbiota enterotypes in 134 lactating dairy goats, distinguished by differences in microbial diversity, community structures, composition, and co-occurrence patterns. These enterotypes were associated with divergent gut fermentation patterns, serum biochemical parameters, and lactation performance in dairy goats.

The TR-cluster exhibited enrichment of *Turicibacter*, *Romboutsia*, and members of Clostridiaceae and Erysipelotrichaceae. *Turicibacter* has been linked to host lipid metabolism, VFA production, and inflammatory modulation (17, 18), with prior evidence showing that its enrichment accelerates growth in young goats (13). *Romboutsia* is known for efficient carbohydrate utilization and VFA production (19, 20). These bacteria may contribute to the enhanced gut fermentation and energy harvest observed in TR-cluster goats. In contrast, the CO-cluster was enriched with genera, such as *Christensenellaceae R-7 group* and *Oscillospiraceae UCG-005*. Both taxa are often associated with lean host phenotypes (21, 22). Additionally, *Christensenellaceae R-7 group* and *Oscillospiraceae UCG-005* are also found to be involved in the fermentation of complex carbohydrates and associated with VFA production (23–25). However, in the present study, their abundance was associated with lower VFA concentrations and reduced lactation performance. This discrepancy may reflect functional redundancy or compensatory metabolic pathways within the CO-cluster microbiome, where these taxa maintain gut microbial stability but may not provide the same energy yield efficiency as those in the TR-cluster. Metatranscriptomic studies are needed to assess whether these taxa exhibit functional plasticity under different conditions.

TR-cluster goats exhibited higher milk yield and milk fat yield, suggesting improved lactation performance. These improvements in production traits were accompanied by increased concentrations of gut VFA, key energy substrates in ruminants (26, 27). The TR-cluster’s enriched VFA production (acetate, propionate, and butyrate) and elevated serum glucose suggest enhanced carbohydrate fermentation efficiency. Propionate probably fuels hepatic gluconeogenesis (28), supporting lactose synthesis, while butyrate promotes mammary acetate utilization for the synthesis of medium-chain fatty acid (C10:0, C14:1c9) (29, 30) and systemic glucose allocation for milk production (31). Elevated serum bile acids in the TR-cluster, potentially driven by bile salt hydrolase activity from *Clostridium sensu stricto 1* and *Turicibacter* (17, 32), may further enhance lipid digestion and milk fat synthesis. Concurrently, higher fecal ammonia nitrogen suggests active microbial protein metabolism supporting milk synthesis. Taken together, these findings collectively suggest that gut microbial enterotypes play a pivotal role in influencing gut fermentation and production performance.

In the present study, the correlation analyses between gut microbiota, gut fermentation parameters, serum biochemistry, and milk production traits further supported these associations, emphasizing the critical role of specific bacterial genera in modulating lactation performance in dairy goats. PICRUSt2 predictions further support this niche specialization. The TR-cluster was enriched in pathways related to energy metabolism, lipid metabolism, and carbohydrate metabolism, consistent with the observed higher milk production and VFA concentrations. In contrast, the CO-cluster was enriched in microbial pathways related to membrane transport, nucleotide metabolism, and cell motility.

The functional attributes of key microbial taxa within the gut ecosystem are crucial in modulating animal health and production performance (33–35). Co-occurrence network analysis revealed that the TR-cluster had a more complex and interconnected microbial network. The greater number of inter-module links in the TR-cluster suggests a tightly coordinated microbial ecosystem, which may facilitate efficient substrate utilization and metabolic outputs beneficial to the host (36). In contrast, the CO-cluster exhibited a sparser network with fewer highly connected nodes, potentially indicating a less cooperative microbial environment. A study in dairy cows found differential microbial interaction patterns between the animals with different feed efficiency, with high-efficiency individuals showing more robust associations than low-efficiency animals (37). Moreover, some taxa, such as *Ruminococcus*, *Christensenellaceae R-7 group*, and *Turicibacter*, were significantly different between the two enterotypes and served as keystone taxa of microbial networks in the gut. Changes in these keystone species could lead to functional shifts in the microbial network, potentially influencing gut fermentation efficiency and, consequently, the production performance of dairy goats.

Several limitations should be acknowledged. First, while 16S rRNA gene sequencing provides valuable taxonomic insights, it offers limited resolution at the species or strain level and cannot capture functional activities directly. Metagenomic or metatranscriptomic approaches would provide deeper insights into the functional capabilities of these enterotypes. Second, while our multi-omics integration provides mechanistic insights, causal links require validation through fecal microbiota transplantation or targeted dietary interventions. Future studies integrating multi-omics approaches, such as metagenomics, metabolomics, and host transcriptomics, are warranted to further elucidate the intricate networks linking the rumen microbiota to host metabolic regulation. Such insights could pave the way for microbiota-targeted strategies to enhance dairy goat productivity.

In summary, this study identifies two distinct gut microbiome enterotypes in lactating dairy goats, characterized by divergent microbial composition, metabolic functionality, and ecological network interactions. These enterotypes were strongly associated with variations in gut fermentation, host metabolic profiles, and lactation performance. Some specific bacterial taxa, including *Turicibacter*, *Romboutsia*, and *Christensenellaceae R-7 group*, and their associations with other bacteria may play important roles in these processes. Our findings highlight the critical role of gut microbiome enterotype in modulating dairy goat productivity. Future strategies could involve dietary interventions or microbiome-directed probiotics tailored to promote efficient enterotypes, thereby optimizing nutrient utilization and milk synthesis efficiency.

## MATERIALS AND METHODS

### Animals, diets, and sampling

A total of 134 healthy, first-parity Guanzhong dairy goats were from a commercial goat farm in Shaanxi, China. Throughout the experimental period, all animals were housed together under identical environmental conditions and received the same management practices. Goats were fed a standardized total mixed ration three times daily (07:30, 13:00, and 19:00) and had *ad libitum* access to feed and water. The ingredients and nutritional components of the diet are shown in Table S1. None of the goats had been treated with antimicrobial agents (including antibiotics, antifungals, or antivirals) or exhibited symptoms of infectious diseases for at least three months prior to the study. All animal procedures were conducted in accordance with the guidelines of the Administration of Affairs Concerning Experimental Animals (Ministry of Science and Technology, China, revised 2004) and were approved by the Institutional Animal Care and Use Committee of Northwest A&F University.

All goats were milked twice daily at 06:30 and 16:00. The milk yield was recorded every seven days throughout the entire first lactation period. Milk samples were collected between the 18th to the 22nd week of lactation for milk composition analysis. Daily milk samples were prepared by combining two-thirds of the morning milk with one-third of the afternoon milk. The average milk yield and composition of each goat during this period were used for subsequent analyses.

During the 23rd week of lactation, blood and fecal samples were collected from all goats. Blood samples were collected via jugular venipuncture into endotoxin-free evacuated tubes, approximately 0–1 h before morning feeding. Samples were centrifuged at 3,500 × *g* for 15 min at 4°C to separate the serum, which was then stored at −80°C until further analysis.

Rectal feces were collected from each goat. To account for diurnal variation, sampling was conducted over a three-day period at 3-hour intervals, ensuring comprehensive coverage of a 24-hour feeding cycle. All samples from each goat were pooled, thoroughly mixed, and immediately snap-frozen in liquid nitrogen.

### Milk composition and fatty acid assay

Milk fat, protein, and lactose concentrations were determined using a MilkoScan FT120 analyzer (Foss Electric, Hillerød, Denmark). Milk fatty acid profiles were analyzed following the method described by Wang et al. (38), with a slight modification. In brief, approximately 0.5 g of freeze-dried milk sample was subjected to direct methylation with 4 mL of 0.5 mol/L sodium hydroxide in methanol at 50°C for 15 min. This was followed by the addition of 4 mL of hydrochloric acid/methanol solution (5% [vol/vol]) and incubation at 50°C for 1 h. The extract was dissolved in 2 mL of heptane and then introduced into a gas chromatograph (Agilent Technologies 7820A GC system, Santa Clara, CA), equipped with a fused silica capillary column (SP-2560, 100 m × 0.25 mm × 0.2 mm; Supelco Inc., Bellefonte, PA).

### Determination of VFA and NH_3_-N in fecal samples

The concentrations of VFA, including acetate, propionate, butyrate, valerate, isobutyrate, and isovalerate, were quantified using a gas chromatography (7820A GC system, Agilent Technologies, Santa Clara, CA, USA), equipped with a flame ionization detector and a fused silica column (AE-FFAP, 30 m × 0.25 mm × 0.33 mm; Agilent Technologies). Approximately 0.5 g of thawed fecal sample was homogenized with 1 mL of distilled water and centrifuged at 13,500 × *g* for 10 min at 4°C. The resulting supernatant (2 mL) was mixed with 500 µL of 25% metaphosphoric acid, followed by centrifugation at 10,000 × *g* for 15 min at 4 °C. Subsequently, 200 µL of crotonic acid solution (10 g/L [wt/vol]) was added to 1 mL of the clarified supernatant. The mixture was filtered through a 0.45 µm organic membrane before being injected into the GC system for analysis. The concentration of NH_3_-N in the fecal supernatant was quantified using a continuous flow analyzer (SKALAR San++, Skalar Co., Netherlands).

### Measurement of serum parameters

Serum concentrations of glucose and blood urea nitrogen were analyzed using the respective commercial assay kits (glucose: Cat. No. A154-1-1; blood urea nitrogen: Cat. No. C013-2-1; Jiancheng Bioengineering Institute, Nanjing, China), according to the manufacturer’s protocols. Glucose concentration was determined by the glucose oxidase peroxidase method following the protocol described by Yuen et al. (39). Blood urea nitrogen concentration was determined using an enzymatic urease glutamate dehydrogenase method as outlined by Kaplan (40). In addition, serum albumin, globulin, total protein, total bile acid, total cholesterol, triglyceride, HDL-C, and LDL-C were analyzed using a fully automated biochemical analyzer (Cobas c311, Roche, Basel, Switzerland).

### Microbial DNA extraction and 16S rRNA gene sequencing

Total DNA in fecal samples was extracted using the QIAamp DNA Stool Mini Kit (QIAGEN, Germany) according to the manufacturer’s protocol. DNA concentration and purity were assessed using a NanoDrop 2000 spectrophotometer (Thermo Fisher Scientific, Waltham, MA), and integrity was checked in 1% agarose gel electrophoresis. The bacterial 16S rRNA gene fragments (V3–V4) were amplified from the extracted DNA using the forward primers 338F (5′-ACTCCTACGGGAGGCAGCAG-3′) and the reverse primer 806R (5′-GGACTACHVGGG T W TCTAAT-3′). All amplicons were sequenced using the paired-end (2 × 300 bp) method on a MiSeq platform (Illumina, USA) according to standard protocols (41).

### Illumina sequencing data analysis

Raw paired-end reads were merged using FLASH (v1.2.11) (42) and the quality filtered with fastp (0.19.6) (43). Amplicon sequence variants (ASVs) were denoised and inferred using the DADA2 pipeline integrated within QIIME2 (version 138) (44). Taxonomic classification of representative ASV sequences was performed against the SILVA 138 reference database.

Microbial alpha diversity indices (ACE richness, Chao1 richness, Sobs, and Shannon diversity index) were calculated at the ASV level with QIIME2. Beta diversity was assessed based on Bray-Curtis dissimilarities, and microbial community structure was visualized using PCoA. The significance of community separation between groups was evaluated using analysis of similarities (ANOSIM) with 999 permutations.

To predict the function profiles of the microbial communities, phylogenetic investigation of communities by reconstruction of unobserved states 2 (PICRUSt2, https://github.com/picrust/picrust2) was employed (45). Predicted metagenomic functions were subsequently annotated and categorized into Kyoto Encyclopedia of Genes and Genomes (KEGG) pathways at level 2.

### Construction of microbial co-occurrence networks based on random matrix theory

Microbial co-occurrence networks were constructed for TR-cluster and CO-cluster using a random matrix theory (RMT)-based pipeline with default parameters following established methodologies (46, 47). Briefly, ASVs detected in <60% of samples within each cluster (TR: *n* = 43; CO: *n* = 91) were excluded to enhance topological reliability (48). The abundance matrix was subjected to Pearson correlation analysis to generate a similarity matrix, from which statistically significant correlations were identified based on the RMT-determined threshold. A fast-greedy modularity optimization algorithm was applied to partition the network into distinct modules. The topological role of each node within the network was evaluated by calculating two parameters: the within-module connectivity (Zi) and the among-module connectivity (Pi). Nodes were classified into four categories based on their Zi and Pi values: peripheral nodes (Zi ≤2.5, Pi ≤0.62), connectors (Zi ≤2.5, Pi >0.62), module hubs (Zi >2.5, Pi ≤0.62), and network hubs (Zi >2.5, Pi >0.62) (49). The networks were visualized using Cytoscape software (v3.8.0).

### Enterotype clustering

Enterotype classification was conducted based on the bacterial community composition profiles at the genus level. Jensen-Shannon divergence was calculated on the genus-level relative abundance data to construct a distance matrix, which was then subjected to partitioning around medoid clustering for enterotype identification (50, 51). The optimal number of clusters was determined using the CH index, with higher CH values indicating better clustering performance. To visualize microbial community structures among samples, PCoA was performed based on Bray-Curtis dissimilarities.

### Statistical analysis

All goats were categorized into TR-cluster and CO-cluster based on their gut microbiome enterotypes. Independent samples *t*-tests were used to compare milk yield, fat-corrected milk yield, milk composition, milk fatty acids, serum biochemical parameters, fecal VFA, and NH_3_-N between the two enterotypes. Differences in microbial taxonomic composition, alpha diversity indices, and predicted functions were assessed using the Wilcoxon’ rank-sum test, with *P* values adjusted by the false discovery rate correction. To construct microbial co-occurrence networks within each enterotype, genera with a relative abundance greater than 0.5% and detected in more than 50% of samples were selected. Significant co-occurrence events were defined as Spearman’s rank correlations with *P* < 0.05. Network visualization was performed using Cytoscape v3.7.1. Associations between gut microbiota and phenotypic variables (milk yield, milk composition, fecal VFA, and serum parameters) were evaluated using Spearman’s rank correlation test, and *P* < 0.05 was considered significant. All data were expressed as the means ± SE. Differences were statistically significant at *P* < 0.05.

## Data Availability

The data sets supporting the results of this article are included within the article and its additional supplemental material. The raw sequence data were submitted to NCBI Sequence Read Archive (SRA) with accession number PRJNA1256928.

## SUPPLEMENTAL MATERIAL

Table S1 spectrum.01367-25-s0001.docx Feed ingredient and chemical composition of dairy goats.

ASM does not own the copyrights to Supplemental Material that may be linked to, or accessed through, an article. The authors have granted ASM a non-exclusive, world-wide license to publish the Supplemental Material files. Please contact the corresponding author directly for reuse.
