# Supplementary material for: Gut microbiome enterotypes drive divergent lactation performance in dairy goats through host-microbe metabolic cross-talk
Source: Microbiol Spectr. 2025 Sep 26;13(11):e01367-25. doi: 10.1128/spectrum.01367-25 (PMC12584760; doi:10.1128/spectrum.01367-25)
Supplement: Table S1 — Feed ingredient and chemical composition of dairy goats. [file spectrum.01367-25-s0001.docx]

**Table S1. Feed ingredient and chemical composition of dairy goats**

| Ingredients, % DM | Content | Chemical composition | Content |
| --- | --- | --- | --- |
| Alfalfa hay | 18.8 | DM, % | 55.20 |
| Corn silage | 31.2 | CP, % DM | 13.95 |
| Corn | 26.8 | Starch, % DM | 30.23 |
| Wheat bran | 13.3 | NDF, % DM | 35.05 |
| Soybean meal | 7.4 | ADF, % DM | 21.88 |
| NaHCO_3_ | 0.4 |  |  |
| CaHPO_4_ | 0.4 |  |  |
| NaCl | 1.6 |  |  |
| Premix^1^ | 0.1 |  |  |

^1^Premix contained (per kg) the following: Cu, 2,925 mg; Fe, 3,900 mg; Zn, 2,750 mg; Mn, 800 mg; vitamin A, 1,500 kIU; vitamin D_3_, 500 kIU; and vitamin E, 5,500 IU. DM = dry matter; CP = crude protein; NDF = neutral detergent fiber; ADF = acid detergent fiber.
